# Prevalence of pancreatitis in UK Miniature Schnauzers: Insights from an owner survey-based study

**DOI:** 10.1186/s40575-026-00155-4

**Published:** 2026-07-09

**Authors:** Arielle Johnson-Pitt, Cathryn S. Mellersh, Collette Taylor, Brian Catchpole, Lucy J. Davison

**Affiliations:** 1 Department of Clinical Science and Services, The Royal Veterinary College, Hertfordshire, AL9 7TA UK; 2 Canine Genetics Centre, University of Cambridge, Cambridge, CB3 0ES UK; 3 Department of Pathobiology and Population Sciences, The Royal Veterinary College, Hertfordshire, AL9 7TA UK; 4 Department of Physiology, Anatomy and Genetics, University of Oxford, Oxford, OX1 3PT UK

**Keywords:** Canine, Pancreatitis, Miniature Schnauzer, Diabetes mellitus, Epidemiology, Hyperlipidaemia, Survey, Owner.

## Abstract

**Background:**

Veterinary epidemiology studies of the Miniature Schnauzer have revealed high prevalence of several breed-associated diseases. However, owner-reported disease data has not been evaluated, particularly with respect to pancreatic diseases, for which this breed is considered predisposed. This study aimed to examine Miniature Schnauzer health from the owner perspective, and explore relationships between reported pancreatitis and other conditions.

**Methods:**

An online survey open to UK-based owners of Miniature Schnauzers in 2023, including directed disease-specific questions and opportunity for free-text reporting. Analyses were performed using R, including multivariable logistic regression modelling.

**Results:**

Responses from 4,786 owners were received, of which 2,910 contained sufficient data for analysis. The most prevalent diseases reported were categorised as “dermatological”, “mass-associated” and “allergic”. Pancreatitis was reported in 7.6% of dogs. A reported diagnosis of pancreatitis was also associated with increased odds of a diabetes mellitus diagnosis. Hyperlipidaemia was reported in 1% of all Miniature Schnauzers, increasing to 3.3% of dogs over the age of 9 years.

**Conclusions:**

This study identifies a high owner-reported prevalence of pancreatitis in UK Miniature Schnauzers, differing from previous veterinary epidemiological studies. The findings highlight the value of incorporating owner-reported data into breed health research and underscores the importance of effective communication between clinician and owner.

**Supplementary Information:**

The online version contains supplementary material available at https://doi.org/10.1186/s40575-026-00155-4.

## Plain Language

Previous studies of Miniature Schnauzer health have relied on veterinary clinical records. However, the owner-perspective has not been explored in detail. We aimed to investigate health conditions in UK Miniature Schnauzers using a survey, with a particular focus on diseases related to the pancreas. Miniature Schnauzers are thought to be prone to diseases of the pancreas such as pancreatitis (inflammation of the pancreas) and diabetes mellitus (a condition where blood sugar is not controlled effectively). Interestingly, some recent large veterinary studies have not reported high levels of pancreatitis in this breed. This raised the question of whether pancreatic disease might be under-recognised, recorded differently in clinical practice, or perceived differently by owners. We therefore wanted to understand how commonly owners report this condition and whether it is linked to other diseases. In 2023, we distributed a survey to owners of Miniature Schnauzers, which included “Yes/No”-style questions and an opportunity to contribute additional clinical information using free-text responses. We analysed the data using statistical methods enabling us to assess the frequency of certain conditions and look for possible associations between them. Overall, the most common diseases reported were skin, allergic, and mass-related conditions, which is very similar to previous studies. However, 7.6% of dogs were reported to have been diagnosed with pancreatitis, which is higher than previous estimates. Additionally, dogs reported to have pancreatitis were also more likely to have diabetes mellitus, suggesting a relationship between these two conditions of the pancreas. We also discovered a lower reporting of hyperlipidaemia (elevated fat levels on the blood) than expected. This study provides new insight into Miniature Schnauzer health from the owner perspective. It highlights differences between owner-reported and veterinary-recorded disease frequencies. The study also suggests that combining both perspectives may improve understanding of breed health and support better communication between veterinarians and owners.

**Supplementary Information:**

The online version contains supplementary material available at https://doi.org/10.1186/s40575-026-00155-4.

## Background

First established as a breed in 1888 [23], the Miniature Schnauzer has enjoyed enduring popularity, and ranks in the top 20 canine breeds in the US [6]. Miniature Schnauzers have increased susceptibility to certain diseases and a large-scale epidemiological assessment of Miniature Schnauzers from UK primary care practices reported periodontal disease, enteropathies, and cutaneous disease as the most common disorders [17]. However, despite recognised predisposition to pancreatic diseases such as pancreatitis and diabetes mellitus in the breed [4, 7, 11, 17, 22, 26, 27], these conditions were not reported in that primary care study. This might be explained by reduced prevalence of these conditions in primary care practice, compared to the referral populations described in other studies, or it could relate to challenges associated with disease recognition. 

The primary care study did report vomiting as a fine-level precision disorder in Miniature Schnauzers (5.1% prevalence), so it is possible that a proportion of those dogs recorded with “vomiting” might represent undocumented or undiagnosed cases of pancreatitis. This is consistent with reports that only 20.7% of clinical problems receive a definitive diagnosis in veterinary practice [20], and could reflect lack of sensitivity and specificity in clinical testing for pancreatitis. The survey presented here provided an opportunity to corroborate whether owners of UK Miniature Schnauzers perceived pancreatic disease to be an issue.

The aim of the current study was to survey owner-reported medical conditions in Miniature Schnauzers in the UK, including specific owner-perceived prevalence of pancreatic disease in this breed. We also sought to evaluate the relationship between pancreatitis and other diseases in this cohort, through use of statistical modelling methods. The availability of online survey tools has enabled owners to participate more directly in studies of pet health and breed-associated conditions. With online survey techniques, researchers can reach a large population of respondents, creating a substantial dataset of information, which is complimentary to clinical data held in electronic patient records. We hypothesise this approach would identify gaps that could be addressed in preventative medicine, veterinary care, or client education and communication.

## Methods

Ethical approval was obtained from the Department of Veterinary Medicine’s Ethics and Welfare Committee (University of Cambridge) (CR607). An online survey was open to UK-based owners of Miniature Schnauzers for 1 month (May 2023), and was advertised and distributed through breed clubs, social media, and newsletters. Survey questions for which data are reported here are listed in Supplementary data S1. Content included closed-style specific-health and phenotype questions and open-style questions with the option for free-text reporting.

The survey gave the opportunity for owners of one or more Miniature Schnauzers to respond to the questions irrespective of life stage. Each survey entry corresponded to a single animal; owners with multiple animals completed a separate survey response for each animal. Identifying personal data were not included in the analysis. Neuter status was recorded at the time of survey. To identify potential outliers arising from data entry errors, bodyweight was screened for extreme values, with Tukey’s fences used to exclude outliers that indicated an error in data entry or a value outside the expected range for the breed (Q3 + 1.5 × IQR). Animal age was calculated as the interval between reported date of birth and survey completion date. For some entries this calculated ages that were implausible for the breed (e.g. ≥ 17 years). As the survey did not collect information on whether the animal was alive at the time of survey completion, it was not possible to determine whether these entries reflected accurate retrospective reporting. Consequently, age was treated as unreliable for these entries and they were excluded from age-based analyses. Respondents who began the survey but did not progress beyond the first question were also excluded from the analysis. Owners who did not finish the survey were excluded from modelling analysis. Where data were missing from individual questions, this was coded as “not recorded” and included in analysis. No other exclusion criteria were applied, in order to capture a broad and unbiased response from participants.

### Tools used

Data were cleaned and coded using Microsoft Excel [14], and parsed, analysed, and visualised using R [19] through the graphical interface R studio [18]. The following packages were used in R: tidyverse, readxl, lubridate, tidyr, finalfit, epiDisplay, dplyr, pROC, ggplot2, pscl, broom, kableExtra, flextable, forestplot, grid, car.

### Analysis of free text

Qualitative content analysis was used to analyse free text, with a thematic-driven coding frame derived from unique codes established though reading a sub-set of responses. These codes were then applied to the remaining responses. Codes were iteratively reassessed and similar codes were grouped into broader categories. Where disorders had already been reported in specific question analysis, these were discounted in free-text to avoid reporting a single patient with a condition multiple times.

### Multivariable regression modelling

Pancreatitis was selected for modelling as it was the disease group with the highest reported prevalence in the population. Modelling was undertaken using a case group (reported pancreatitis diagnosis, *n* = 220) versus a control group (no reported pancreatitis diagnosis, *n* = 2459). Explanatory variables identified at the univariable level (*p* < 0.2) were included in the multivariable logistic regression model, with variables showing high collinearity excluded. Where variables with 3 levels (“yes”, “no”, “not recorded”) reported only a significant (*p* < 0.05) level of “not recorded” these were excluded from multivariable modelling as this likely reflected bias from missing data. These variables were excluded even if the Likelihood Ratio Test (LRT) comparing model fit yielded a *p*-value < 0.2.

A manual forwards stepwise approach was used in multivariable model building with most-least significant variables sequentially added to the model. Variables were retained in the model if the likelihood ratio test remained significant (*p* < 0.05) and the AIC value reduced when compared to the null model.

Pairwise interactions between all explanatory variables were assessed using logistic regression models including the interaction term, with significance evaluated by likelihood ratio tests comparing models with and without the interaction. Model fit to the dataset was assessed using the Likelihood Ratio Test and pseudo R^2^. Model predictive performance was evaluated with a receiver operative characteristic (ROC) curve, with the area under the curve (AUC) used to quantify discriminative ability.

## Results

Of the 4,786 respondents, 2,910 responses were of adequate quality in terms of data completeness to progress with the analysis, with 2,669 responders completing the entire survey (8.3% is an acceptable degree of ‘missingness’ to not require adjustment for bias [1]). Data completeness from analysis of the 2,910 responses was: sex 98.2%, neuter status 98.0%, date of birth 94.4%, Body Condition Score (BCS) 95.7%, bodyweight 83.0%. For the 2,910 owner-responses containing information for the appropriate variable, there were 1,347 (47.1%) females, and 2,105 dogs (73.8%) were reported to be neutered (Table 1). Median age was calculated as 5.5 years. The most commonly reported BCS was 3/5, or “ideal”, by 2,030 (72.9%) respondents. This was divided equally between males and females. For a BCS of 4/5, there were 159 (5.7%) dogs reported, and for BCS 5/5, 17 (0.6%) dogs, indicating an owner-reported percentage of obese/overweight Miniature Schnauzers of 6.3% (n = 176). Adjusted mean bodyweight was 9.2 kg (SD 1.9 kg). Males had a reported mean bodyweight of 9.9 kg (SD 1.8 kg), which was heavier than females at 8.5 kg (SD 1.7 kg).

**Table 1:** Frequency of known and unknown sex and neuter status reported by Miniature Schnauzer owners

	Entire	Neutered	Unknown neuter status
Female	228 (7.8%)	1109 (38.1%)	10 (0.3%)
Male	518 (17.8%)	972 (33.4%)	19 (0.7%)
Unknown sex		24 (0.8%)	30 (1.0%)

Free text data were available from 1,638 responses. This was assessed using a thematic-driven coding frame established from 40 unique codes constructed from reading a sub-set of 200 responses. These codes were then applied to the remaining 1,438 responses. Codes were iteratively re-assessed and similar codes were grouped into 22 categories (Table 2). Age results are reported here as median [range].

**Table 2:** Codes and categories derived from free-text responses

Codes for free-text	Category	Total reported (*n*=)
allergy_food	Allergic	229
allergy_misc	Allergic	
atopy	Allergic	
alopecia	Dermatological	289
dermal_cyst	Dermatological	
dermal_misc	Dermatological	
pruritus	Dermatological	
schnauzer_comedone_syndrome	Dermatological	
anal_gland	Anal gland	184
behaviourial	Behavioural	14
cardiac	Cardiac	11
ccl_rupture	Musculoskeletal	117
osteoarthritis	Musculoskeletal	
patellar_luxation	Musculoskeletal	
other_orthopaedic	Musculoskeletal	
cryptorchid	Developmental abnormalities	37
umbilical_hernia	Developmental abnormalities	
congenital	Developmental abnormalities	
crystalluria	Lower urinary tract	17
cystitis	Lower urinary tract	
urolithiasis	Lower urinary tract	
dental_disease	Periodontal	210
dermal_mass	Mass-associated	286
internal_mass	Mass-associated	
epilepsy	Neurological	41
neuropathy	Neurological	
gastroenteritis	Gastrointestinal (specific)	111
haemorrhagic_gastroenteritis	Gastrointestinal (specific)	
other_gastrointestinal	Gastrointestinal (specific)	
gb_mucucoele	Hepatobiliary	16
hepatic_shunt	Hepatobiliary	
hepatobiliary_disease	Hepatobiliary	
hepatopathy	Hepatobiliary	
misc	Other	43
other_endocrine	Other	
non_specific_gastrointestinal	Gastrointestinal (non-specific)	62
onychodystrophy	Claw or nail	15
otitis_externa	Aural	74
renal_disease	Renal	6
trauma	Trauma	15

Free-text responses (*n* = 1638) were assigned to 40 codes developed from a subset of 200 responses. Similar codes were grouped into 22 categories. “Total reported (n)” indicates the number of responses coded under each category

**Fig. 1:**
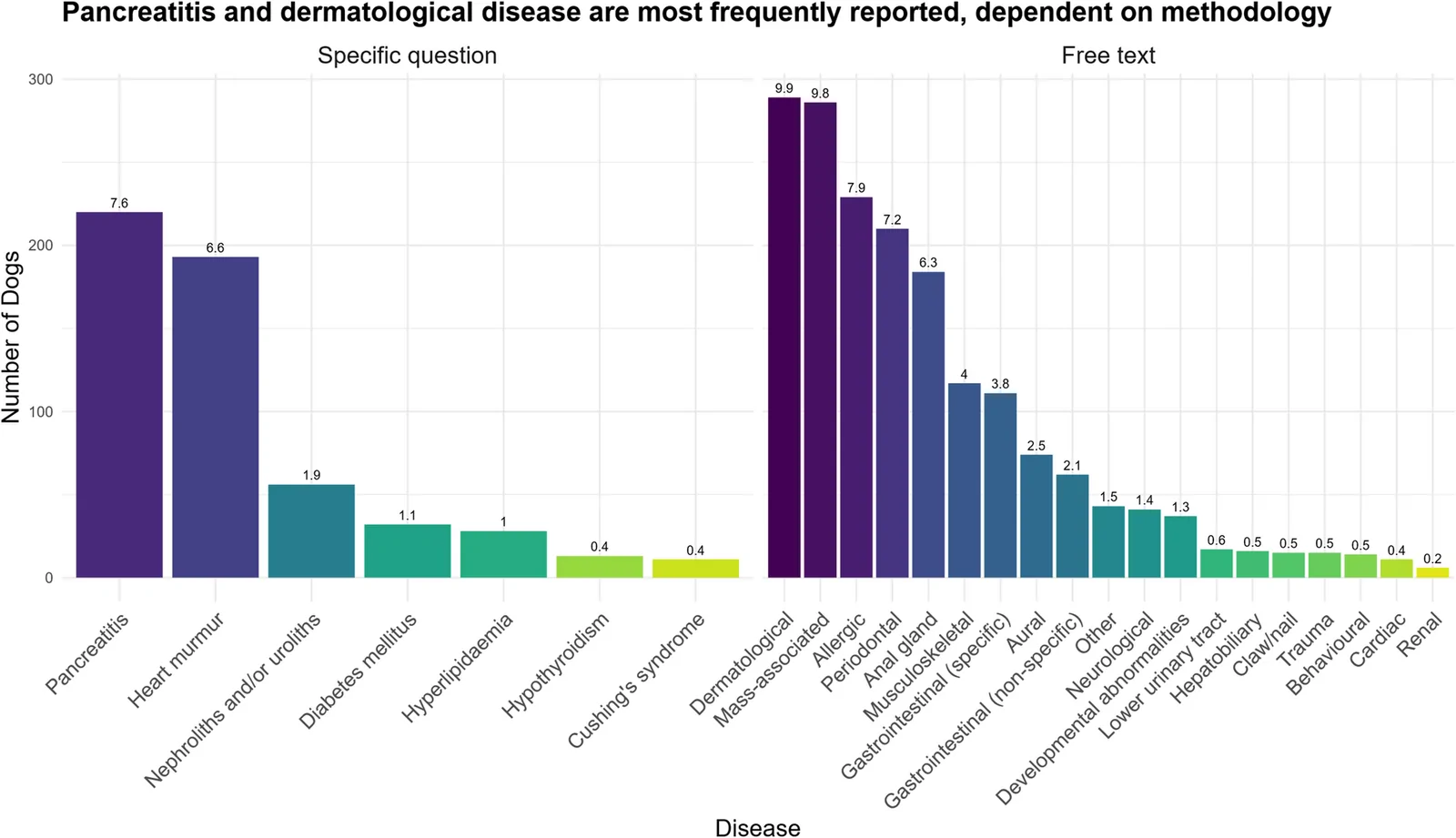
Disease frequency reported by Miniature Schanuzer owners via survey response. Compares disease reports through responses to specific question format with “yes” / “no” answers for seven diseases and free text anaylsis (grouped into disease categories). Percentages above represent the proportion of 2,910 respondents

**Fig. 2:**
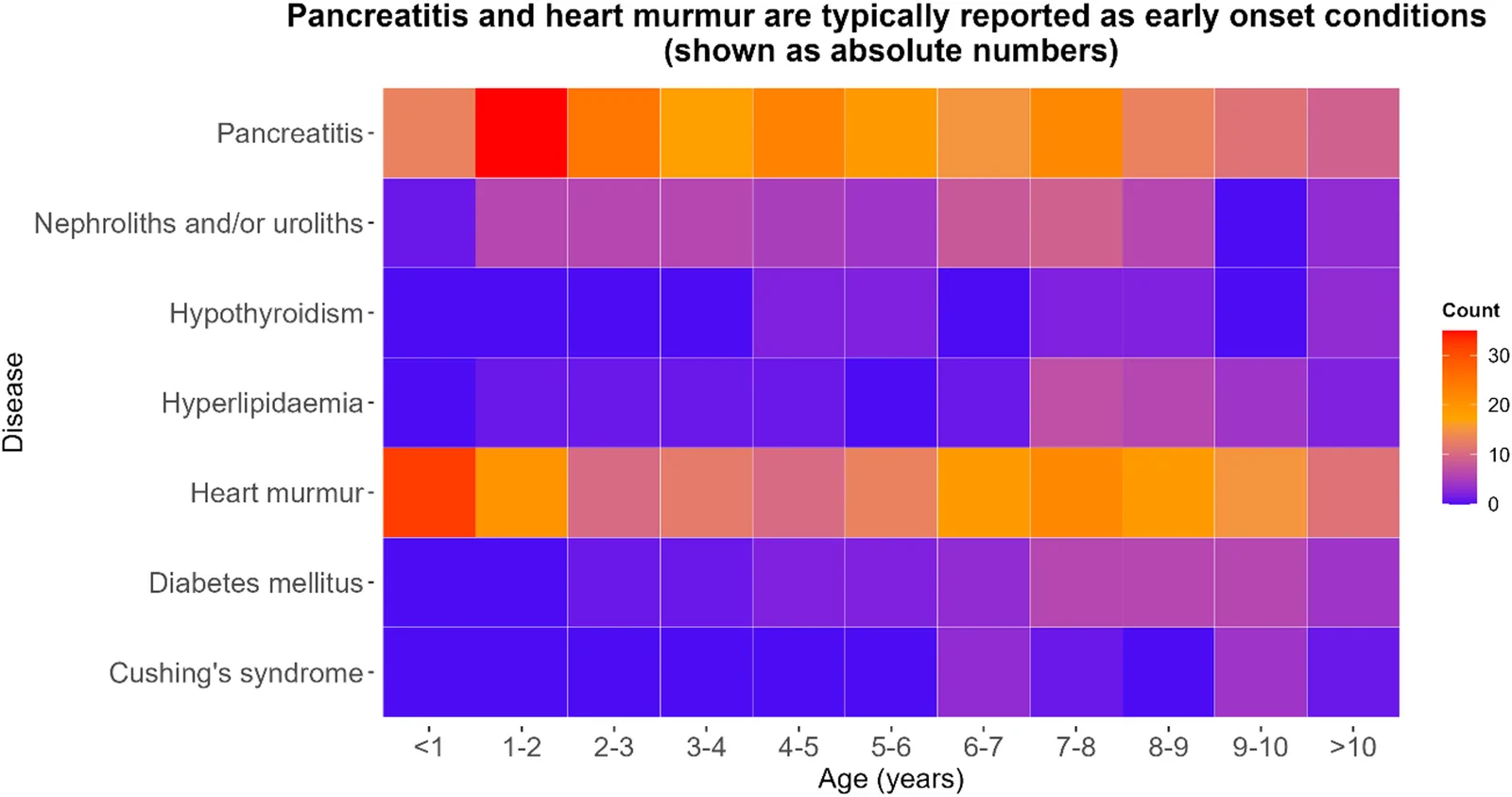
Heatmap illustrating responses to the “age of onset” question format across seven specified diseases. Colours represent response distribution, highlighting variation in reported onset ages for each disease

For specific disease questions (Fig. 1), pancreatitis was reported by owners in 220 (7.6%) of animals, heart murmur in 193 (6.6%), nephroliths and / or uroliths in 56 (1.9%), diabetes mellitus in 32 (1.1%), hyperlipidaemia in 28 (1.0%), hypothyroidism in 13 (0.4%), and hyperadrenocorticism (Cushing’s syndrome) in 11 (0.4%). Where age at diagnosis was reported for heart murmur, 21% (33/257) were diagnosed under the age of 1 year, and where age at diagnosis was reported for pancreatitis, 17.2% (35/203) were diagnosed between the ages of 1 and 2 years. Of this pancreatitis subpopulation, there were 13 females and 22 males, of which 9 were entire (8 male and 1 female); the remainder were neutered. Pancreatitis cases with concurrent diagnosis of diabetes mellitus had similar age of diagnosis of both diseases, with 7/12 patients reporting the same age of diagnosis for both diseases, and the remainder reporting that pancreatitis was diagnosed 2 years after a diagnosis of diabetes mellitus, although there was no significant difference in age of diagnosis of the two conditions (*p* = 0.83, Wilcoxon rank-sum test). Notably, compared to hyperlipidaemia (7.5y [1y-12y]), pancreatitis (4y [5 m-14y]) had an earlier age of diagnosis (*p* < 0.0001, Wilcoxon rank-sum test) (Fig. 2). Twelve dogs had diagnoses of both pancreatitis and hyperlipidaemia, with 9 reporting age of onset for both conditions. Four of 9 cases reported the same age of onset, with the remaining 5/9 reporting diagnosis of pancreatitis prior to diagnosis of hyperlipidaemia. There were 16 reported dogs with a diagnosis of hyperlipidaemia but not pancreatitis. In dogs diagnosed with both pancreatitis and hyperlipidaemia, age at diagnosis did not differ significantly between the two diseases (*p* = 0.06, paired Wilcoxon signed-rank test).

## Multivariable regression model

To understand any relationship between pancreatitis and other independent variables, a multivariable regression model was designed. Sixty-seven variables were assessed in univariable analysis. Of these, 17 were liberally significant (LRT *p*-value < 0.2) and taken forward for analysis. Collinearity was evaluated using the variance inflation factor (VIF); variables identified as having aliased coefficients were not included simultaneously in the same model. This included age at neutering and neuter status (“entire” category was colinear) and anti-lipaemic medication and gastrointestinal medication (where the “not recorded” category was colinear, as these variables were derived from free-text analysis).

**Fig. 3:**
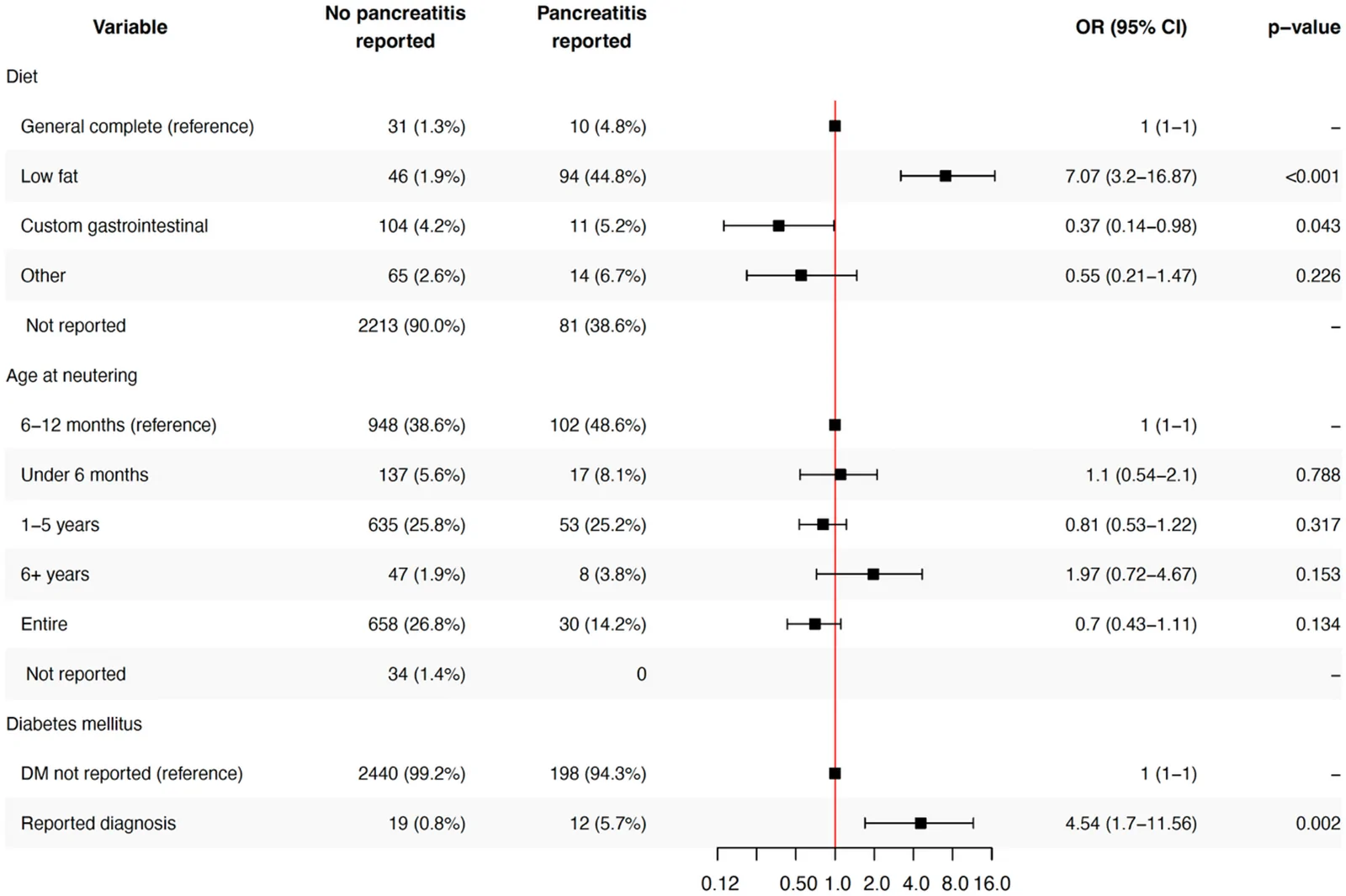
Forest plot illustrating results of multivariable regression model comparing Miniature Schnauzers with a reported diagnosis of pancreatitis to those without a reported diagnosis of pancreatitis

To improve the stability of the model, diet was collapsed into 4 groups: general complete (which served as the reference), low fat, custom gastrointestinal, and “other”. Similarly, the data reporting age at neutering was collapsed, forming categories of 6–12 months (reference), < 6 months, 1–5 years, 6 + years, and entire (Fig. 3). Interactions between diabetes mellitus and other variables were explored; however, due to the small number of cases in the diabetes group, interaction terms were not included in the final model.

The final model contained 3 variables: diabetes mellitus, diet, and age at neutering. A reported diagnosis of pancreatitis was associated with increased odds of a diabetes mellitus diagnosis (OR = 4.54, 95% CI: 1.7–11.6, *p* = 0.002) and was also associated with being fed a low-fat diet (OR = 7.07, 95% CI: 3.2–16.9, *p* < 0.001). The variables ‘age at neutering’ and ‘diet’ significantly improved model fit (LRT *p* < 0.05). For the ‘age at neutering’ variable, no specific age groups was significantly associated with a reported diagnosis of pancreatitis, whereas a ‘low-fat diet’ was significantly associated with this diagnosis.

The likelihood ratio test was significant comparing fitted to null, (*p* < 0.001) and AIC values were smaller than the null model AIC, indicating the associated variables collectively improved model fit. McFadden’s pseudo-R^2^ was 0.27, indicating good model performance. The ROC analysis calculated an AUC value of 0.80 (95% CI: 0.77–0.84), suggesting good discrimination between outcome groups.

## Discussion

Understanding owner perception of their pets’ health is an important aspect of veterinary medicine. This study revealed a high prevalence of owner-reported pancreatitis in the Miniature Schnauzer breed, with some cases having an early onset of disease at 1–2 years of age. There was also a clear relationship between pancreatitis and diabetes mellitus, with diabetes typically occurring at the same time, or after recognition of pancreatitis.

In general, owner-reported findings were broadly consistent with veterinary reported disease in the Miniature Schnauzer breed, and in the broader canine population [16, 17]. The most prevalent conditions were dermatological, mass-associated, and allergic disease. However, the prevalence of pancreatitis reported by owners of Miniature Schnauzers here (7.6% of respondents) was higher than reported in primary care studies [17], [17]. This finding, however, must be interpreted with caution, given the challenges and complexity of diagnosing pancreatitis and the lack of clinical evidence available to verify owner reports.

The relationship between the presence and timing of a pancreatitis diagnosis and other conditions was also explored. Whilst concurrent diseases such as hyperlipidaemia, diabetes mellitus, and hypercortisolism are known to predispose to secondary pancreatitis in older dogs ([13, 24]), it was unexpected here that 17.2% (35/203) of owners described the onset of disease between 1 and 2 years of age, indicating a primary rather than secondary origin to the pancreatitis. Chronic pancreatitis can result in exocrine pancreatic insufficiency (EPI), although there were no reports of EPI cases in the survey responses when analysing the free-text data.

It is also recognised that pancreatitis can predispose to endocrine pancreatic disease, such as diabetes mellitus [9, 15]. Here, the reported prevalence of diabetes mellitus in this Miniature Schnauzer population was 1.1%. This is higher than the reported general canine population prevalence of 0.26% − 0.38% [3, 12], and consistent with previous studies reporting increased diabetes risk in the breed ([2, 27]). Furthermore, dogs reported as having diabetes mellitus had 4.54 times the odds of a reported pancreatitis diagnosis (95% CI: 1.7-11.56, *p* = 0.002). The issue of whether exocrine pancreatic inflammation precedes diabetes mellitus or arises from uncontrolled diabetes mellitus is contentious in dogs, and although the former appears more common, it is likely that either can occur [5, 9]. In this survey, where owners reported an age of diagnosis for concurrent diabetes mellitus and pancreatitis, they were typically either diagnosed at the same time, or with pancreatitis arising prior to diabetes. Long-term follow-up of the cases of pancreatitis reported here would be valuable in determining what proportion of Miniature Schnauzers with pancreatitis develop diabetes mellitus with increasing age.

An increased risk of hyperlipidaemia is also recognised in the Miniature Schnauzer breed. This typically develops later in life and increases in severity and complexity with age [25, 26]. A study from the USA reported that > 75% of Miniature Schnauzers over the age of 9 have hypertriglyceridaemia, with an overall breed prevalence of 32.8%. In the present study, median reported age of diagnosis was 7.5 years, consistent with these previous observations [26] and in dogs diagnosed with both hyperlipidaemia and pancreatitis, the age at diagnosis did not differ significantly between the two diseases. This survey indicates an owner-reported prevalence of hyperlipidaemia of only 3.3% in Miniature Schanuzers over the age of 9 years. Based on other prevalence studies reported above, this is considerably lower than would be expected. This might reflect lack of testing, or could relate to genetic or environmental differences in the UK and USA populations. Dietary factors including high-fat diet, or dietary indiscretions [8, 11], have also been associated with pancreatitis risk, but other, unascertained environmental factors might also exist. Dietary modification in the form of a low-fat food, is often recommended for management of hyperlipidaemia and / or pancreatitis and this is likely to explain the observed increased odds ratio for being fed a low-fat diet in dogs with a pancreatitis diagnosis.

## Limitations of presented data

An important limitation of these results is the lack of veterinary input into reporting of conditions: the testimony of owners might not accurately reflect the true presence or absence of confirmed clinical disease. In particular, owners and vets are very likely to differ in their specific criteria for a pancreatitis diagnosis. In studies of human patients, variable concordance between self-reported conditions versus analysis of biomedical data is evident [10]. In veterinary medicine, it is recognised that bias can be introduced by factors such as poor recall of clinical information, or poor comprehension of reported clinical diagnoses, dependent on the complexity of the condition being described [21]. Here, reports of more straightforward diagnoses such as diabetes mellitus with specific medication (exogenous insulin administration) might be more reliable than more complex diagnoses of diseases such as pancreatitis or hypercortisolism. To strengthen the reliability of the findings, future research would benefit from direct comparison between clinician-reports and owner-reports. It was also unknown whether any dogs had visited a referral centre, which may influence study findings, as diagnoses in such cases might more accurately reflect the full range of clinical conditions present in each dog. Furthermore, results might be subject to reporting bias, with owners of dogs affected by one or more clinical conditions more likely to complete the survey than owners of healthy dogs. Additionally, the findings may be influenced by reporting bias, as owners of affected dogs may have been more motivated to participate than owners of clinically healthy animals. Participation was further limited to individuals reached through the survey distribution pathways, potentially introducing selection bias. When exploring dogs reported to have pancreatitis, and those with pancreatitis and diabetes, it is important to acknowledge that these were only small proportions of the dataset (*n* = 12 dogs in total with both diseases), highlighting the need for further targeted studies in a larger population of Miniature Schnauzers. These small numbers might also explain the lack of reporting in the previously mentioned large-scale studies. A particular limitation of survey-style research is the potential influence of question framing, including leading questions; however, participants were provided with opportunities for free-text responses to encourage more comprehensive and nuanced input. Despite these limitations, these results do provide some valuable insights into disease pathogenesis. A notable strength of this study compared to studies based on electronic health record data is the availability of detailed information which is not routinely captured in larger-scale datasets, such as time of neutering and age of disease onset. The availability of these data will assist in informing and refining further research questions.

As the data were time-restricted and only represented owner recall within three months in 2023, there is also a degree of missingness to the data, for example where younger animals, currently healthy, may later develop more typically geriatric pathology (such as diabetes mellitus). Nonetheless, use of time-restricted data can have valuable utility as a cross-sectional “snapshot” of a breed’s characteristics and allows a baseline for trend analysis to be used in future studies. In future surveys, it would also be beneficial to reduce the number of free-text responses to facilitate comparative analyses, understand how disease diagnoses were determined, and potentially reduce the length of the survey: a factor which might have contributed to incomplete responses from some participants.

## Conclusion

This study demonstrates a discrepancy between primary care documented diagnosis of pancreatitis in the Miniature Schnauzer breed, and relatively high owner reporting of the condition, which requires further investigation. We also report an increased odds ratio of diabetes mellitus in Miniature Schnauzers with pancreatitis, suggesting a relationship between the two diseases. The relatively early age of onset of pancreatitis in some Miniature Schnauzers also raises the possibility of a primary disease in these dogs rather than pancreatitis secondary to other conditions. Comprehension of owner perspective is increasingly valuable in an interconnected world and future studies should focus on aligning findings in clinical records with owner perceptions in the same dogs, to better inform patient treatment and welfare.

## Supplementary Information

Below is the link to the electronic supplementary material.

Supplementary Material 1

## Data Availability

The datasets generated and analysed during the current study are not publicly available due to privacy and confidentiality considerations relating to owner-reported data but data are available from the corresponding author on reasonable request.
