# Supplementary material for: Prevalence of pancreatitis in UK Miniature Schnauzers: insights from an owner survey-based study
Source: Companion Anim Health Genet. 2026 Jul 9;13:6. doi: 10.1186/s40575-026-00155-4 (PMC13349035; doi:10.1186/s40575-026-00155-4)
Supplement: Supplementary file 1 — Supplementary Material 1 [file 40575_2026_155_MOESM1_ESM.docx]

MS survey

Start of Block: Questions about your Miniature Schnauzer

Q1
Thank you for participating in this survey that is being undertaken by the Kennel Club Genetics Centre at the University of Cambridge, in collaboration with veterinary ophthalmologists Christine Heinrich (Eye Veterinary Clinic) and internal medicine experts Lucy Davison (Royal Veterinary College) and Eleanor Raffan (University of Cambridge).

Questions 2, 3, 4 are excluded as they form part of a separate study

Q5 Questions about your Miniature Schnauzer

Q6 Q1. Is your Miniature Schnauzer Kennel Club registered?

- Yes (1)
- No (2)
- Don't know (3)

Display This Question:

If Q1. Is your Miniature Schnauzer Kennel Club registered? = Yes

Q7 What is his/her KC registered name, if known?

________________________________________________________________

Display This Question:

If Q1. Is your Miniature Schnauzer Kennel Club registered? = Yes

Q8 What is his/her KC number, if known

________________________________________________________________

| 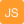 |
| --- |

Q9 Q2. When was your Miniature Schnauzer born? If you don't know their exact date of birth please enter the approximate year they were born

|  | Month | Day | Year |
| --- | --- | --- | --- |
|  |  |  |  |
| Please Select: (1) | ▼ January (1 ... December (12) | ▼ 1 (1 ... 31 (31) | ▼ 1900 (1 ... 2049 (150) |

Q10 Q3. Is your Miniature Schnauzer male or female?

- Male (1)
- Female (2)

Q11 Q4. If your Miniature Schnauzer has been neutered or spayed at what age was this done?

- (s)he has not been spayed or neutered (1)
- Under 6 months (2)
- 6-12 months (3)
- 1 year old (4)
- 2 years old (5)
- 3 years old (6)
- 4 years old (7)
- 5 years old (8)
- 6 years old (9)
- 7 years old (10)
- 8 years old (11)
- 9 years old (12)
- 10 years or older (13)
- I don't know (14)

End of Block: Questions about your Miniature Schnauzer

Questions 12 to 20 are excluded as they form part of a separate study

Start of Block: Questions about your Miniature Schnauzer's weight/body condition

Q21 Questions about your Miniature Schnauzer's weight/body condition

Q22 Q6. Do you know how much your dog weighs?

- Yes (1)
- No (2)

Display This Question:

If Q6. Do you know how much your dog weighs? = Yes

Q23 Please enter your dog's weight in the box below, in kg

________________________________________________________________

| 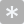 |
| --- |

Q24 We would like to know about your dog's body condition score.

Below is a graphic demonstrating the scores, along with the descriptions. Please select the score that most closely resembles your dog.

|  | Off (1) | On (2) |
| --- | --- | --- |
| bcs_1 (7) |  |  |
| bcs_2 (8) |  |  |
| bcs_3 (9) |  |  |
| bcs_4 (10) |  |  |
| bcs_5 (11) |  |  |


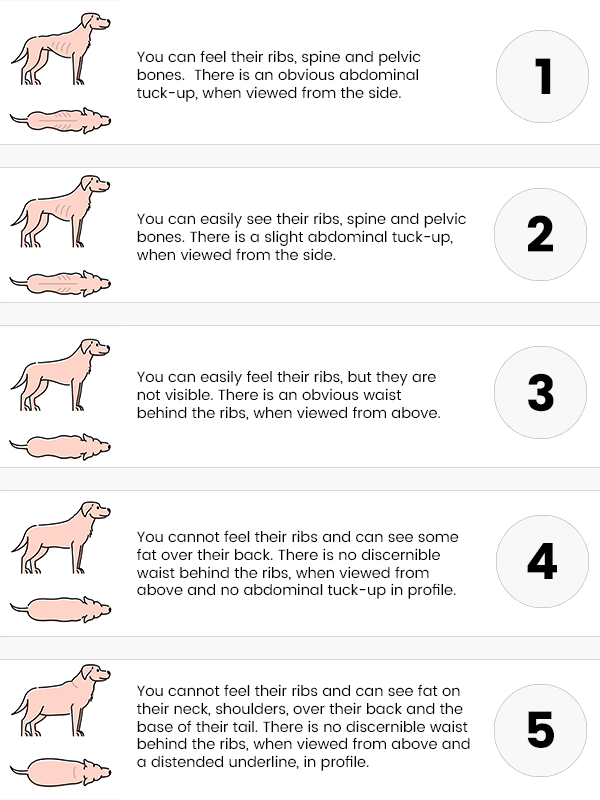


End of Block: Questions about you Miniature Schnauzer's weight/body condition

Start of Block: Questions about other health conditions

Q25 Questions about other health conditions

Q26 Q7. Has your Miniature Schnauzer ever been diagnosed with any of the following (tick all that apply):

- Pancreatitis (1)
- Abnormal blood lipids/fats (dyslipidemia) (2)
- Cushing's disease (3)
- Diabetes mellitus (requiring insulin treatment) (4)
- Hypothyroidism (underactive thyroid) (5)
- Kidney or bladder stones (6)
- Heart murmur (7)
- None of the above (8)

Display This Question:

If Q7. Has your Miniature Schnauzer ever been diagnosed with any of the following (tick all that app... != None of the above

Q27 For each condition please tell us how old your dog was when they were diagnosed

- Pancreatitis (1) __________________________________________________
- Abnormal blood lipids/fats (dyslipidemia) (2) __________________________________________________
- Cushing's disease (3) __________________________________________________
- Diabetes mellitus (requiring insulin treatment) (4) __________________________________________________
- Hypothyroidism (underactive thyroid) (5) __________________________________________________
- Kidney or bladder stones (6) __________________________________________________
- Heart murmur (7) __________________________________________________

Q28 Q8. Is your Miniature Schnauzer on any medications, a special diet or any supplements?

- Yes (1)
- No (2)

Display This Question:

If Q8. Is your Miniature Schnauzer on any medications, a special diet or any supplements? = Yes

Q29 Please tell us what they are taking and how long they have been taking them for.  Don't worry too much about spelling, just indicate the medication by name or tell us what it is for

- Medications (1) __________________________________________________
- Special diet (2) __________________________________________________
- Supplements (3) __________________________________________________

Q30 Please use the box below to tell us anything else about the health of your Miniature Schnauzer – either now or in the past - that we haven’t already asked about. Examples here might include skin allergies, heart rhythm problems, dental disease, arthritis, problems with anal glands, operations, skin lumps

________________________________________________________________

________________________________________________________________

________________________________________________________________

________________________________________________________________

________________________________________________________________

Q31 Would you be willing for us to contact you again in the future to request a DNA sample from your Miniature Schnauzer?  The DNA can be collected using a simple cheek swab that you can take yourself at home and returned to us via regular mail?

- Yes (1)
- No (2)

Display This Question:

If Would you be willing for us to contact you again in the future to request a DNA sample from your... = Yes

Q32 Please tell us your name (optional)

________________________________________________________________

Display This Question:

If Would you be willing for us to contact you again in the future to request a DNA sample from your... = Yes

Q39 Please tell us your email address so that we can contact you in the future

________________________________________________________________

End of Block: Questions about other health conditions
